# Multi-Omics Approaches for the Prediction of Clinical Endpoints after Immunotherapy in Non-Small Cell Lung Cancer: A Comprehensive Review

**DOI:** 10.3390/biomedicines10061237

**Published:** 2022-05-26

**Authors:** Vincent Bourbonne, Margaux Geier, Ulrike Schick, François Lucia

**Affiliations:** 1 Radiation Oncology Department, Regional University Hospital, 29200 Brest, France; ulrike.schick@chu-brest.fr (U.S.); francois.lucia@chu-brest.fr (F.L.); 2 LaTIM, UMR 1101 INSERM, University of Brest, 29200 Brest, France; 3 Medical Oncology Department, Regional University Hospital, 29200 Brest, France; margaux.geier@chu-brest.fr

**Keywords:** immune checkpoint inhibitor, non-small cell lung cancer, prediction, personalized medicine

## Abstract

Immune checkpoint inhibitors (ICI) have revolutionized the management of locally advanced and advanced non-small lung cancer (NSCLC). With an improvement in the overall survival (OS) as both first- and second-line treatments, ICIs, and especially programmed-death 1 (PD-1) and programmed-death ligands 1 (PD-L1), changed the landscape of thoracic oncology. The PD-L1 level of expression is commonly accepted as the most used biomarker, with both prognostic and predictive values. However, even in a low expression level of PD-L1, response rates remain significant while a significant number of patients will experience hyperprogression or adverse events. The dentification of such subtypes is thus of paramount importance. While several studies focused mainly on the prediction of the PD-L1 expression status, others aimed directly at the development of prediction/prognostic models. The response to ICIs depends on a complex physiopathological cascade, intricating multiple mechanisms from the molecular to the macroscopic level. With the high-throughput extraction of features, omics approaches aim for the most comprehensive assessment of each patient. In this article, we will review the place of the different biomarkers (clinical, biological, genomics, transcriptomics, proteomics and radiomics), their clinical implementation and discuss the most recent trends projecting on the future steps in prediction modeling in NSCLC patients treated with ICI.

## 1. Introduction

Despite recent epidemiology changes, lung cancer remains the leading cause of death by cancer and among the most frequent cancers [1]. With approximately 50% of the non-small cell lung cancers being diagnosed at a metastatic stage [2], prognosis remains poor, with a 24-month overall survival (OS) rate reaching only 10–23%, according to the 8th AJCC classification [3].

Immune checkpoint inhibitors (ICI) have revolutionized the management of locally advanced and advanced non-small lung cancer (NSCLC). With an improvement of OS as both first- [4,5,6] and second-line [7,8] treatments, ICIs, and especially programmed-death 1 (PD-1) and programmed-death ligands 1 (PD-L1), changed the landscape of thoracic oncology. As a second line treatment, nivolumab increased the median OS from 9.4 months to 12.2 months [7]. Similar results were observed with pembrolizumab [8]. As a first-line treatment in NSCLC patients with a PD-L1 expression >50%, pembrolizumab alone improved the 6-months OS rate from 72.4% to 80.2% in comparison to chemotherapy alone. Regardless of the PD-L1 expression level, a combination of chemotherapy and immunotherapy increased the 12-months OS rate from 49.4% to 69.2% [9,10,11].

When sub-analyzing the results, the PD-L1 expression and level of expression appeared as the most used biomarker and is now a screening molecular biomarker when considering pembrolizumab as a first-line monotherapy. However, despite a less significative impact of anti-PD-1/PD-L1 agents when considering patients with a PD-L1 expression <1%, the clinical benefit persisted highlighting the insufficiency of PD-L1 expression as a sole biomarker, but also the complexity of the patient’s response to ICIs. Moreover, a significant number of patients will experience hyperprogression [12] or adverse events [13]. The identification of such subtypes is thus of paramount importance [14,15,16,17].

Numerous studies focused on the stratification of patients with regards to their response to ICIs. While several studies focused mainly on the prediction of the PD-L1 expression status, others aimed directly at the development of prediction/prognostic models. The response to ICIs depends on a complex physiopathological cascade, intricating multiple mechanisms from the molecular to the macroscopic level. With the high-throughput extraction of features, omics approaches combined with machine learning approaches aim for the most comprehensive assessment of each patient [18]. In this article, we will review the place of the different biomarkers (clinical, biological, genomics, transcriptomics, proteomics and radiomics), their clinical implementation and discuss the most recent trends projecting on the future steps in prediction modeling in NSCLC patients treated with ICIs.

## 2. PD-L1 Expression: The Historical Biomarker

Tumoral cells historically share similar features, among which immunosuppression is one of the main resistance mechanism to the immune system [19]. The PD-1/PD-L1 pathway regulates the activation of tumor-infiltrating lymphocytes (TILs). The binding of the PD-1 receptor localized on the surface of activated T-cells to one of its ligands (PD-L1) blocks the T-cell activation and subsequent immune responses, leading to a poorer prognosis [15,20,21]. The inhibition of this binding via anti-PD-1 or anti-PD-L1 drugs could thus restore the T cells’ functions, resulting in significant clinical benefits in several clinical settings such as melanoma and NSCLC.

In the NSCLC setting, anti-PD-1 drugs were first introduced as a second-line treatment with a significant benefit on OS over chemotherapy, with the level of PD-L1 expression as low as 1% [7,8]. Focusing on the sub-set of patients with a PD-L1 expression >50%, pembrolizumab significantly increased both progression-free survival (PFS) and OS [6]. Apart from the Checkmate 057 trial, the Keynote 010 and Keynote 024 had a respective minimum PD-L1 expression level of 1% and 50%. In the Checkmate 057 trial, pre-planned sub-analysis of the results based on the level of PD-L1 expression confirmed the prognostic value of PD-L1 expression [22].

The value of PD-L1 expression was validated with a longer follow-up in several meta-analyses. In an aggregated meta-analysis focusing on five different ICIs (pembrolizumab, nivolumab, atezolizumab, durvalumab and avelumab), the PD-L1-positive patients (cut-offs varying from 1% to 50% depending on the included studies) had a two-fold higher response rate when compared to PD-L1-negative patients, translating into a significantly longer PFS (HR 0.69, 95% CI: 0.57–0.85) and OS (HR 0.77, 95% CI: 0.67–0.89) [23]. The value of the PD-L1 expression remained even when considered as a categorical feature (and not binary) [24] and further validated on a patient-level meta-analysis [25]. 

Despite these encouraging results, stratification through the PD-L1 expression level remains insufficient. Firstly, the PD-L1 expression level is often evaluated on a biopsy sample (either the primitive or a metastatic site) in patients with, sometimes, numerous metastatic sites. The PD-L1 expression level is known as heterogenous among the disease site [26], but also can change after certain treatments [27]. While the heterogeneity of expression could explain the dissociated responses, the second point raises issues on the risk of anti-PD-1/anti-PD-L1 resistance [28]. A certain variability was also seen regarding the methodology for the PD-L1 expression assessment, with some considering the tumor proportion score (TPS) and others the combined proportion score (CPS) [29]. The TPS is evaluated as the percentage of PD-L1-positive tumor cells over total tumor cells. The CPS is the percentage of all PD-L1-stained tumor cells, lymphocytes and macrophages compared with that of all tumor cells [15]. Moreover, the PD-L1 expression level does not present the occurrence of hyperprogression in a substantial number of patients (5–20%), irrespective of the PD-L1 expression level, nor does it prevent rapid relapse in PD-L1 > 50% patients [12]. To this day, PD-L1 expression remains the most used biomarker for ICIs in NSCLC patients and leads to major therapeutic differences between PD-L1 > 50% and <50% patients, with only the first subset having the possibility of first-line monotherapy with pembrolizumab and the latter being treated with either chemotherapy or a combination of ICIs and chemotherapy. New biological biomarkers such as Tumor Mutational Burden (TMB) or the neutrophils-to-lymphocytes ratio were thus evaluated and could complement the expression level of PD-L1 and better understand patients’ response to ICIs [30].

## 3. The Contenders: TMB, TILs, Neutrophils-to-Lymphocytes Ratio

With 1833 patients included in the individual patient-level analysis, PD-L1 expression and tumor mutation burden (TMB) were associated with several clinical endpoints [25]. Based on a whole-exome sequencing, TMB achieved an AUC of 0.84 for the prediction of a 3-year PFS. When focusing on targeted loci, the AUC rose to 0.95 with no PFS benefit from ICIs over chemotherapy for low TMB patients. OS appeared as significantly different between high and low TMB patients (HR 0.26, *p* = 0.009), in line with other studies, suggesting the value of TMB as a powerful biomarker [31,32]. The assessment of the blood tumor mutation burden (bTMB) has the advantage of a non-invasive approach with much less evaluation. In a small set of 42 patients, bTMB was significantly correlated with progression-free survival [33], conforming with similar results [34,35,36]. DNA methylation also appears as an efficient prediction tool in NSCLC, with the benefit of a better understanding of the underlying molecular determinants [37,38,39].

The response to ICIs depends on the tumor microenvironment, namely tumor-infiltrating lymphocytes (TILs). Often defined as “hot” tumors (higher proportion of high CD8^+^ T-cells), TILs-enriched tumors are more likely to benefit from ICIs, as opposed to “cold” tumors [32,40,41,42]. The small correlation between CD8^+^ TILs and PD-L1 expression or TMB explains the synergy between these three biomarkers for response prediction [25]. TILs are only one mediator among numerous others also involved in the PD-1/PD-L1 signaling pathway and response to ICIs [43]. Acting as either induction or inhibition factors, natural killer cells, monocytes and B-lymphocytes [44] play a major role in the response when stimulated/inhibited by a variety of chemokines and cytokines (TNF-α [45], IFN-γ [46,47,48], interleukines [49,50], cell growth factors [44,51,52,53], etc.). Among other biological mediators, exosomes could also play a role in the induction of the expression of PD-L1 on macrophages [54,55]. Further research is needed to fully understand how exosomes could impact the response to ICIs.

Among more clinical/biological biomarkers, the neutrophils-to-lymphocytes ratio (NLR) appears as a predictive tool across different types of malignancies receiving ICIs. Patients with a high NLR experienced worse OS (HR 1.92, *p* = 0.001) and PFS (HR 1.66, *p* < 0.0001) [56]. Similar results, yet statistically non-significant, were found in NSCLC patients (HR 1.63 for OS). NLR has the great advantage to be easily accessible via a blood sample when compared to TMB, for example. Based on derived NLR (dNLR) and lactate deshydrogenase (LDH), the Lung Immune Prognostic Index (LIPI) was predictive of PFS and OS, but only in patients treated with ICI (and not chemotherapy), suggesting the specificity of the LIPI for ICI response prediction [57,58,59,60]. NLR was used in several other models. Combining NLR with other clinical/biological features (performance status, dNLR, liver metastases, smoking status and LDH) identified three prognostic groups for PFS prediction [61]. The EPSILoN score was externally validated with promising results even on OS prediction (HR 2.40, *p* < 0.001) [62]. Focusing on patients included in phase 1 trials, a model combining NLR, LDH and albumin levels was prospectively validated in a cohort of patients undergoing ICI-based therapies in phase 1 trials [63] and further externally validated [64].

The majority of these biomarkers provide a global appreciation of the response to ICIs without really measuring its underlying mechanics. Going to the molecular level could maybe provide a better assessment and understanding of the response to ICIs.

## 4. Genomics, Transcriptomics and Proteomics: Understanding the Cascade

Given the numerous steps involved in the response to ICIs, using genomics, transcriptomics and proteomics as biomarkers opposes itself to the multiplicity of signaling pathways to explore and the difficulty to conjugate all the findings.

A genomics signature combining 13 different genes through a random forest reached an AUC of 1.0 for the prediction of a durable clinical response (DCB), significantly higher than the high PD-L1 expression, or the high TILs/TMB levels [65]. The Tumor Inflammation Signature (TIS) is another genomics signature that measures a pre-existing, but suppressed adaptive immune response within tumors. This 18-gene signature was validated within the Cancer Genome Atlas (TCGA) with multiple represented histologies, among which were 980 lung cancer patients [66]. Specific genes such as KLRG1, BTK, CCR2 and SCML4 were associated with poor responses to immunotherapy [67]. ART1 expression, for example, drives ICI resistance by promoting CD8^+^ T-cell death and could be a therapeutic target [68]. Other genes (JAK1/JAK2) also appear as potential targets to restore the efficacy of ICIs [69]. Mismatch repair deficiency/microsatellite instability-high is a predictor of anti-PD-1/PD-L1 therapy efficacy, but remains a niche in NSCLC patients, with less than 1% of affected patients [70]. When considering squamous-cell carcinoma (SCC) and adenocarcinoma (ADK) patients separately, different pathways are identified. For instance, the loss or inactivation of STK11 in ADK patients or KEAP1/NFE2L2 alterations in SCC patients are associated with a reduced immune response. To be noted, neither of these remained significant after adjustment for the expression subtype [71].

Transcriptomics characterize tumors and patients based on extracted ribonucleic acids (RNAs). Several studies have focused on the value of micro RNAs and non-coding RNAs for the prediction of responses to ICIs. Several tissue-extracted microRNAS such as miR-200b and miR-508 appear as positively correlated with the response to ICIs, while others such as miR-429 are negative biomarkers [72]; however, this did not remain significant after multivariate analysis when compared to PD-L1 expression. Based on a large cohort of NSCLC patients, a model combining seven non-coding RNAs significantly stratified patients into immune-cold and immune-hot cohorts, remained independent from other clinical/biological features and could complete the PD-L1 expression as a biomarker [73]. Using a bioinformatics approach, patients with a higher immune cell infiltration score based on the combination of multiple RNAs showed a significantly increased clinical benefit [74]. Interestingly, high ICI scores were associated with the activation of the T-cell and B-cell receptor signaling pathways and natural killer cell–mediated cytotoxicity. Using RNA expression levels and a machine learning approach, gene expression levels were proven to be superior and independent or complementary from PD-L1 expression [75,76]. When developing predictive models, SCC and ADK are often considered together. Discriminant analysis of the immune cells’ signatures in the SCC/ADK patients revealed key differences in the immune host response depending on the histology.

α1-Acid glycoprotein (AGP) is a serum glycoprotein with diverse immunomodulating effects and a huge variety of structures [77]. Glycan structures can change in association with the presence of a tumor or under certain treatment conditions [78,79]. In a small but prospective cohort treated with nivolumab, fucosylated AGP levels appeared a reliable biomarker [80].

One pitfall of such genomics or transcriptomics approaches is the need of tumor sample. Using blood-base biomarkers has a double advantage: the easiness of access, but also the possibility of longitudinal analyses and thus the evaluation under treatment. Circulating cell-free DNA after the first cycle of immunotherapy and a variation of circulating tumor cells between the first and second cycle might be biomarkers for PFS and could change the management of NSCLC patients under ICIs [81].

Most of these biomarkers come with the cost of pricey analyses and additional exams for the patients. Using available exams, such as follow-up computed tomography (CT), positron emission tomography (PET) or magnetic resonance imaging (MRIs), could be as effective without overloading patients with additional blood or tumor samples.

## 5. Radiomics/Deep-Learning: The One to Unite Them All?

Radiomics consist of high-throughput extraction quantitative features from medical imagings, using several parameters (voxel resampling, discretization, eventual filters, etc.). In an effort to facilitate the evaluation of a radiomics-based model, the International Biomarker Standardization Initiative has harmonized the definition of features and defined the mandatory steps for such approaches [82]. 

Radiomics approaches could be divided into two sub-groups: research papers focusing on the prediction of already-known biomarkers and articles aiming directly for the prediction of the clinical endpoint [83].

Several radiomics models were able to predict the level of expression of PD-L1 with reasonable performances [84]. For instance, using a small retrospective overall cohort of 72 patients and combining two features extracted from pre-treatment CTs, the model reached an AUC of 0.79 for the prediction of PD-L1 values ≥ 50% in the validation cohort [85]. Combining three different features resulted in the prediction of PD-L1 values between 1 and 49% with an AUC of 0.81 in the validation cohort. With differences relying mainly on dataset size, methodological choices and clinical endpoints, a variety of radiomics signatures were thus recently published, the majority focusing on the prediction of PD-L1/TMB expression [86,87,88,89,90,91,92] or even the assessment of TILs [93].

The prediction of the PD-L1 value could be presented as of limited interest if restricted to the initial pre-treatment evaluation, with the PD-L1 evaluation being necessarily performed on the tumor sample used for diagnosis. However, it could provide a simple and non-invasive tool for the monitoring of the PD-L1 level of expression under treatment.

Directly targeting the endpoint has the advantage of fully appreciating the complexity of the response to immunotherapy. Using either baseline imagings [94] or longitudinal imagings with delta-radiomics analyses [95,96], several models were proposed with very high performances [97]. With the advantage of prospectively included, but retrospectively analyzed patients treated with nivolumab, a radiomics signature achieved an AUC of 0.77 in the validation dataset [98]. In a pan-cancer analysis, a radiomics signature based on 203 patients with advanced melanoma and NSCLC achieved an AUC of 0.76 for the prediction of immunotherapy response with a 1-year survival difference of 24% between the two risk groups. The correlation between this radiomics signature and the signaling pathways involved in the mitosis suggest the ability of the model at stratifying patients based on their molecular characteristics, but through non-invasive imaging [99].

Pathomics relates to the quantitative analysis of histopathological images. Based on deep learning with an internal validation, a pathomics-based model was able to predict several biomarkers such as TP53 and EGFR mutational status [100]. Bioinformatics pipelines applied to pathology exams were also evaluated with moderate results (AUC 0.65) and were uncorrelated to the TMB [101]. A comparison with the PD-L1 level of expression or TILs was not available in the lung dataset.

**Figure 1:**
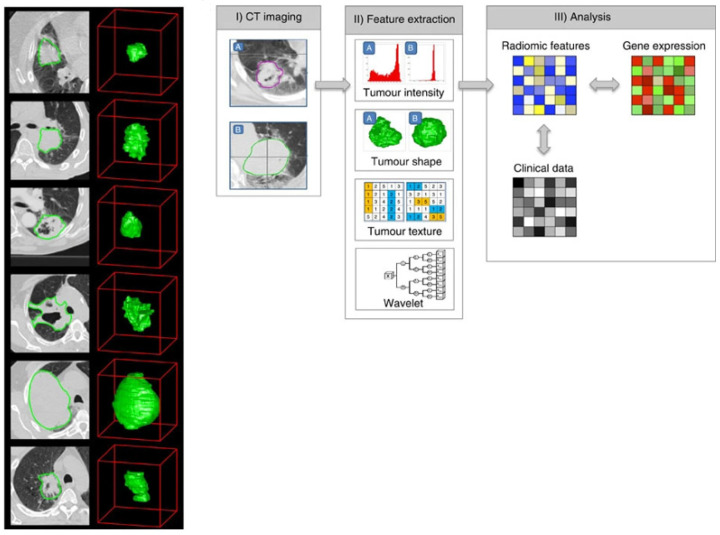
Typical workflow in a radiomics study, reproduced with permission from [104].

In more recent years, deep-learning approaches applied to radiology exams have focused on the same issues. While a radiomics workflow first extracts features from the segmented volume of interest and then combines them (Figure 1), a deep-learning approach directly uses the image or volume of interest as the input.

Such approaches were used for the prediction of PD-L1 expression [90,102], as well as the main clinical outcomes [103].

Despite all addressing similar clinical endpoints, none of the radiomics or deep-learning-based signatures are used on a daily-clinical basis. Similar difficulties can also be found regarding genomics or transcriptomics biomarkers, with only the NLR-based models being really used. Understanding the difficulties underlying the lack of clinical implementation seems necessary for the development and/or validation of future personalized medicine tools.

## 6. Challenges Regarding the Clinical Implementation of Next-Generation Biomarkers

The large majority of previously presented studies were either developed in a small cohort or not validated prospectively and/or in multicentric cohorts. Apart from the prediction signatures relying on clinical and biological features [40,61,62,63,64], only a few were actually externally validated. Given the risk of overfitting and low generalizability using machine learning approaches with small datasets, one can presume the results “to be too good to be true”, especially with AUCs reaching scores as high as 1.0 [65]. Regarding radiomics-based signatures, a Radiomics Quality Score [105] was developed to evaluate each study with several key points, such as the use of a calibration plot or decision curve analysis, prospective/external validation or the comparison between the developed model and already available prediction models. Models were, for the last majority, ranked as low in this clinical setting [106] with a high heterogeneity between methodological approaches. When considering prediction modeling in NSCLC, not comparing with usual features such as PD-L1 expression, age, sex or performance status is debatable [107,108,109]. Tumor stage is particularly troublesome as it is a major clinical predictor of PFS and OS and is often highly correlated with certain radiomics features. The clinical benefit of radiomics signatures without proving their superiority to the clinical stage is insufficient [104,110]. 

Furthermore, the vast majority of these models are based on retrospective data with a high risk of bias, especially in a clinical setting where multiple factors can influence the response to ICIs. For instance, concomitant radiotherapy is known to increase the response to ICIs and has a significant impact on PFS and OS [111,112,113,114,115]. The history of radiotherapy was documented in none of the above-presented prediction studies.

**Figure 2:**
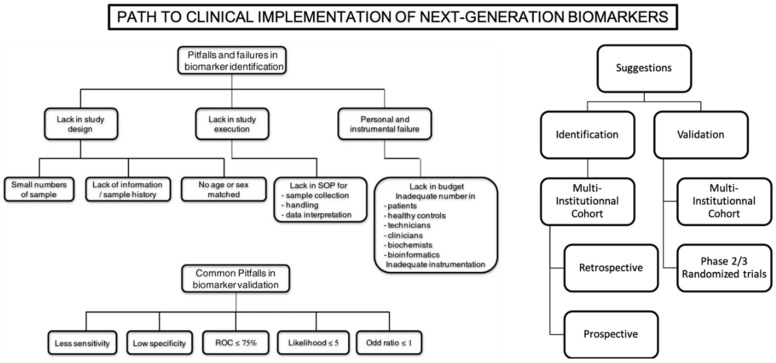
Main limitations and propositions for clinical implementation of next-generation biomarkers. Reproduced and adapted with permission from [122].

Genomics models have a step-forward regarding their clinical implementation across all cancer types, but not in NSCLC. To our knowledge, two genomics tests are widely used: the OncotypeDX for breast cancer patients [116] and the Decipher test for prostate cancer patients. These models stand out from the others thanks to their multicentric and even prospective validation [116,117,118,119,120,121]. To this day, such validation studies lack in the NSCLC setting. One could think the implementation of biomarkers such as TMB could be easier, this approach being limited by the cost and the technical heterogeneity of its assessment among centers. These limitations are the main pitfalls for the true clinical implementation of such biomarkers (Figure 2).

Acknowledging the difficulty of such an extensive data collection, an artificial intelligence framework was proposed, integrating the baseline and longitudinal electronic health records. This approach was robustly associated with mortality among patients with early- or advanced-stage cancers [123]. However, this framework remains limited to the available data. Many uncertainties remain concerning the response pathway to ICIs, with new leads being continually explored. For example, among the more recent discoveries, intestinal microbiota has experienced a growing interest of researchers, both as a mediator to the ICI response and as a therapeutic target. 

## 7. The Microbiome: The New Eden?

Unlike corticosteroids which were closely monitored and even prohibited during the initial pivotal studies, the administration of antibiotics (ATB) for ICIs was less supervised. Gut microbiota is a complex ecosystem essential for maintaining gut homeostasis and preventing systemic inflammation [124,125]. ATBs alter gut microbiota diversity and composition, leading to dysbiosis [126], which may affect effectiveness of ICIs, as shown in several retrospective studies [127]. In a cohort of 239 patients with NSCLC, patients who received ATB within 30 days of beginning ICIs had a significantly shorter PFS (HR 3.1, *p* < 0.01) and OS (HR 3.5, *p* = 0.03) [128]. A greater diversity of the gut microbiota seems to be related to an increased PFS [129], with higher levels of *Akkermansia muniniphilia* in the stools of responders [127]. Gut microbiota is involved in adaptive immunity and especially T cells. The regulation of specific cells such as CD4^+^ T regulatory cells or CD4^+^ T helper cells can be triggered by certain bacteria [130,131], even though signaling pathways underlying these actions remain unclear [132,133,134]. 

While most studies have focused on analyzing the influence of gut microbiota, its composition substantially differs from that in the lung. Gut and lung microbiota are connected thanks to a complex bidirectional axis via lymphatic and blood circulation, with one microbiota possibly impacting the other [135]. Lung microbiota could play a role in the carcinogenesis through chronic inflammation [136,137,138]. Its role in the response to ICIs remains yet to be discovered [139].

Discovering the role of gut microbiota leads to new therapeutical targets [140]. A fecal microbiota transplant (FMT) was developed in order to modulate gut microbiota and enhance the response rate and/or ameliorate toxicity. Most of the trials involving FMT focus on melanoma with very published data in NSCLC patients [141]. Focusing on a single type of bacteria could possibly be the solution, as suggested by a recent phase 1 trial in renal cell carcinoma patients. Bacterial supplementation with CBM588 in combination with nivolumab–ipilimumab increased the median PFS from 2.5 months to 12.7 months [142].

Given its role in the response to ICIs, gut microbiota could also be used as a biomarker. Analyzing and sequencing stool samples before and under treatment could help in the stratification of patients based on their microbiota [143]. A gut environment, enriched in bacteria such as *Bifidobacteria* spp. [144,145], *Akkermansia muciniphilia* [127], *E. hirae* [146] and *Bacteroides* spp. [147] could predict a longer duration of the response to ICIs. Using microbial sequences, a microbiome model was able to discriminate between stage I and IV cancers for certain histologies (colon, gastric and renal cancers), independently from other biomarkers such as genetic alterations [148]. Given these abilities, one can think the microbiome to be an easily accessible and highly informative diagnostic and prognostic tool, which offers the possibility of multiple time measurements and longitudinal follow-up [149]. 

Identifying the individual bacterial species or the ideal gut microbiota environment remains a challenge [150], but it should be taken into account when designing clinical trials or analyzing previous results. 

**Figure 3:**
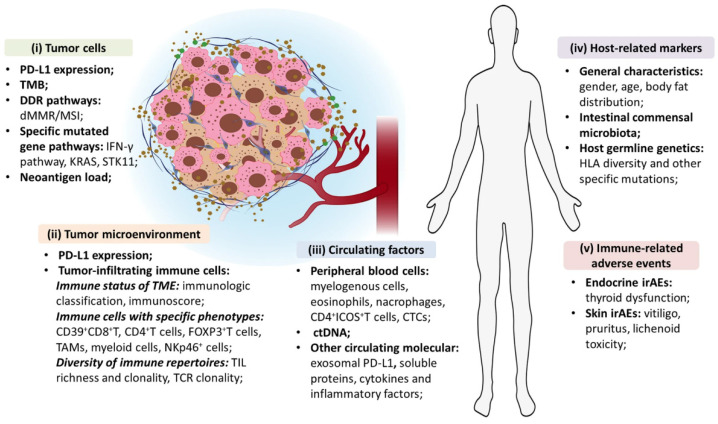
Exhaustive overview of biological biomarkers for the prediction of ICIs’ efficacy in NSCLC patients. Reproduced with permission from [151].

An exhaustive overview of available prediction biomarkers is presented as Figure 3, with only the most relevant being presented in this review.

## 8. Stereotactic Radiotherapy: A Confounding Factor

Stereotactic radiation therapy (SRT) and stereotactic body radiation therapy (SBRT) have seen a tremendous development in recent years. Delivered to either the brain (SRT) or extra-cranial sites (SBRT), it allows a high-dose treatment of small targets with a high conformation, protecting the surrounding organs at risk (OARs). It can be offered in patients with either an early-stage disease or oligo-metastatic or oligo-progressive disease within diverse sites such as the brain, lung and, more recently, the liver, kidney and prostate [152,153,154,155,156,157]. Stereotactic radiotherapy alone is associated with high local control (LC) rates [158,159,160,161,162]. However, until recently, radiotherapy was not expected to be associated with an out-of-field response or have an impact on OS. Surprising out-of-RT field responses were first reported in 1953 by Mole, raising the term of the abscopal effect (from the Latin ab scopus: far from the target) [163]. This phenomenom was defined as the regression of out-of-field metastatic lesions after local radiotherapy [164]. The immuno-modulation of stereotactic radiotherapy could be explained by the complex cascade of biological responses following its delivery, including DNA damage directly on the tumor cells, as well as on the tumor-associated strama and endothelium, with the initiation and circulation of various signal transduction pathways leading to a proinflammatory tumor microenvironnement (TME). As previously presented, this subtype of TME is known to be correlated with a higher response to ICIs [32,40,41,42]. Propsective reports on the synergistic effect of the combination of ICIs and stereotactic radiotherapy remain scarce, the understanding of these complex mechanisms needing further research. Nevertheless, several studies suggest a positive impact of stereotactic radiotherapy on clinical endpoints such as LC and OS rates. For instance, the PEMBRO-RT trial evaluated the benefit of SBRT (24Gy in 3 fractions) at a single site seven days before the initiation of pembrolizumab (vs. pembrolizumab alone) in NSCLC metastatic patients. The median OS was increased from 7.6 months to 15.9 months in the experimental arm [161]. These encouraging results raise several questions, such as the best choice for the site to treat, for the RT protocol or for the ICIs to deliver. The timing of RT delivery regarding the start of ICIs also seems important. Many trials are either enrolling or under analysis, especially in NSCLC patients (NCT03693014, NCT02318771, NCT03867175 or NCT04650490). Given the probable benefit of adding stereotactic radiotherapy to ICIs in selected patients, an evaluation of the biomarkers should take into account the delivery of SRT/SBRT as a possible confounding factor, along with others such as antibiotics and corticosteroids [165].

## 9. Conclusions

As demonstrated, the response to ICIs in NSCLC was often seen as a hodgepodge where multiple features were involved. PD-L1 expression remains the most used biomarker, even if only approaching the surface of prediction modeling. With the concern of the most complete understanding, multiple prediction models involving TMB, TILs, the level of circulating DNAs or miRNAs and even proteomics were developed. Without additional exams, but with their share of limits, radiomics also appeared as promising. To this day, none outperformed PD-L1 expression for clinical implementation because of their heterogeneity and lack of external/prospective validation. Clinicians and researchers should pursue their efforts in aggregating clinical, biological and imaging data in large datasets [166]. More recently, the gut microbiota stood out as a potential diagnostic and prognostic tool, as well as a therapeutic target, with numerous ongoing trials being awaited (NCT04682327, NCT04924374, NCT04954885, NCT05037825 or NCT04638751).
